# Teaching obstetric ultrasound at Mulago Hospital - Kampala, Uganda

**DOI:** 10.4314/ahs.v18i1.21

**Published:** 2018-03

**Authors:** Homa Ahmadzia, Sarah Cigna, Imelda Namagembe, Charles Macri, France Galerneau, Urania Magriples

**Affiliations:** 1 The George Washington University Medical Faculty Associates, Department of Obstetrics and Gynecology, 2150 Pennsylvania Avenue NW, Room 6A 412, Washington, DC 20037; 2 Yale University, New Haven, CT; 3 Makerere University, Kampala, Uganda

**Keywords:** Ultrasound, obstetric, teaching, Uganda, low-resource, curriculum

## Abstract

**Background:**

Mulago Hospital is a high volume referral hospital under the Makerere University School of Medicine and Health Sciences. Basic obstetric ultrasound is a useful skill that can aid patient care.

**Objectives:**

The purpose of the study was to assess the effectiveness of an intervention implemented to teach basic ultrasound skills to medical students and house officers at Mulago Hosptial, Kampala, Uganda.

**Methods:**

Forty participants, including medical students, junior house officers (JHOs), and senior house officers (SHOs) were enrolled in the study. A didactic and practical hands-on teaching session was evaluated using a pre- and post-test that was administered to all participants.

**Results:**

Participants included 12 medical students, 23 JHOs, and 5 SHOs. A significant difference in pre- and post-test scores was demonstrated in the medical students and JHOs (34% to 76%, p <0.0001) and this was retained when the results were stratified into the basic definitions and practical sections of the survey (33% to 71%, p<0.0001). The scores for the senior house officers had a mean increase of 2.3 points.

**Conclusion:**

This original teaching intervention is an effective method to improve knowledge and skills for medical students and house officers at Mulago Hospital in the area of basic obstetric ultrasound.

## Introduction

Uganda is one of 16 landlocked developing countries in Africa, and this results in significant economic disadvantages. Mulago National Referral Hospital is one of three major hospitals in Uganda. It is located on the outskirts of the city center of Mulago and is also a teaching hospital under the Makerere University School of Medicine and Health Sciences. The labor and delivery unit of Mulago Hospital is extremely busy, with up to 35,000 deliveries annually.^1^ Ultrasound, a relatively low cost tool for evaluating obstetric patients, has been found to be particularly useful in low-resource areas of the world.^2^,^3^

Teaching ultrasound skills has previously been studied in underserved areas and developing countries,^2^–^11^ including the evaluation of training of midwives to use ultrasound in obstetrics in rural areas of Uganda. Limited information in the literature is available for describing the actual process and effectiveness of training medical providers to use obstetric ultrasound in low-resource areas. In addition, no teaching or testing material is available in the literature that can be used to carry out the objectives in resource-limited settings. Furthermore, the importance of evaluating and sharing teaching materials and methods is underscored by several studies demonstrating that the primary barrier to effective use of ultrasound in developing countries is the lack of training.^14^,^15^

The purpose of this study was to assess the effectiveness of a teaching intervention implemented to teach basic ultrasound skills, such as identification of fetal lie, placental position, amniotic fluid volume assessment and recognition of fetal breathing and motion, to medical students, interns and residents at Mulago Hospital, Kampala, Uganda.

## Methods

Volunteer medical students, junior house officers (JHOs) and senior house officers (SHOs) greater than or equal to 18 years of age at Mulago hospital were enrolled from mid-November 2010 to mid-December 2010 to participate in a pre-test survey to assess for their basic ultrasound knowledge and practice. IRB approval was obtained from both Yale School of Medicine and Makerere University School of Medicine prior to starting the study. Prior to entering the survey, consent was obtained from all participants. All those who volunteered to participate during the time period studied were enrolled in the study. Exclusion criteria were participants under 18 years of age.

Study protocol broadly included the following: (1) To assess baseline knowledge, attitude and skills in basic obstetric ultrasound for SHOs, JHOs and 5^th^ year medical students. (2) To implement a didactic teaching intervention. (3) To assess post intervention knowledge, attitude and skills. This study involved research on special education instructional strategies. A survey was created (Appendix 1.A), containing questions covering three main categories: (I) participant demographics and self-assessment of ultrasound proficiency, (II) knowledge of basic definitions, and (III) practical knowledge such as visual skills for fetal and amniotic fluid measurements, fetal placentation and placental location. The survey was administered before and after the educational intervention. Correct answers of the survey and participant scores of the pre-test were not disclosed until after the post-test was administered. Each participant was assigned a code to identify the pre/post survey.

The teaching intervention consisted of a combination of didactic education followed by hands-on demonstration of skills, for a total of approximately two hours. Topics of the short instructional course included basic obstetric principles including fundamentals of performing an ultrasound and optimizing an image, fetal presentation, placental position, amniotic fluid index, fetal biometry and biophysical profile. Skills were taught in a separate, hands-on interactive portion for two to three participants at a time with demonstration using patient volunteers with ongoing pregnancies in the second trimester. The didactic and hands-on training were provided by one trainer (HKA) who at the time was a third year OB/Gyn resident with senior level experience in basic obstetric ultrasound. The post-test survey was conducted immediately following the hands-on session. De-identified data (including assigned ID code, age, sex and number of years of training) was collected in Microsoft Excel and analysis was performed in Excel and SAS version 9.2. Paired t-test values were used for pre-post test scores and a p-value of <0.05 was used for statistical significance. The number of participants needed for 90% power to detect a 40% difference in pre and post-test survey results was 25.

## Results

A total of 40 participants underwent the instructional and hands on portions of the intervention. This group included a mixture of 12 medical students, 23 JHOs, and 5 SHOs with reported prior training in obstetric ultrasound for 25%, 81%, and 100% of their groups, respectively (63% with prior training experience overall), Table 1. Overall the percentage of participants with any comfort performing basic obstetric ultrasound at pre- and post-test increased from 23% to 73%. Prior to the intervention, 8% of the medical students, 24% of JHOs and 60% of SHOs reported any comfort with performing basic obstetric ultrasound. Post-intervention surveys demonstrated an increase in post-test comfort for all three groups.

**Table 1:** Prior experience and comfort level of participants prior to and post the training.

	Age	SD	% prior training	% any comfort pre	% any comfort post
MS (n=12)	23	0.7	25	8	67
JHO (n=23)	26	1.7	81	24	81
SHO (n=5)	31	2.0	100	60	80
**N=40**	**26**	**2.7**	**63**	**23**	**73**

Evaluation of the participants' proficiency in the fundamentals of basic obstetric ultrasound is demonstrated in Table 2. Across levels the overall pre-post difference was 5.8 (95% confidence interval 4.8 – 6.8), which was a significant increase (p<.0001). There was a significant difference between pre- and post-test scores in the medical student and JHO groups; mean difference in raw score was 5.3 and 4.9 points, respectively (p <0.0001).

**Table 2:** Differences in pre and post-test scores of all participants.

Level	Mean Score Pre (SD)	Mean Score Post (SD)	Mean difference, SD	95% CI	p value
Medical Student (n=12)	5.8 (2.4)	12.2 (1.1)	6.8 (2.3)	5.3 to 8.2	<0.0001
Junior House Officer, JHO (n=21)	5.0 (2.4)	11.3 (2.1)	6.1 (2.7)	4.9 to 7.3	<0.0001
Senior House Officer, SHO (n=5)	9.4 (1.5)	11.0 (0.8)	1.5 (2.4)	−2.3 to 5.3	0.30

The pre- and post-test scores for the senior house officers had a mean increase of 2.3 points; however this was not statistically significant. Statistical significance was retained in the medical student and JHO group score differences when the results were stratified into the basic definitions and practical skills sections of the survey (p <0.0001) (see Table 3). The SHO group showed no improvement in the knowledge component of the survey and minimal improvement in the practical component (mean score difference 1.00).

**Table 3:** Differences in pre and post-test scores broken down by section of questions asked on survey.

	Section*	Mean Difference Post — Pre (95% CI)	Score p value
Medical Student (n=12)	2	3.25	<0.0001
		(2.56–3.94)	
	3	3.17	<0.0001
		(2.21–4.13)	
Junior House Officer,	2	2.90	<0.0001
JHO (n=23)		(2.30–3.51)	
	3	3.24	<0.0001
		(2.31–4.16)	
Senior House Officer, SHO (n=5)	2	0.00	1.0
		(−2.12 – 2.12)	
	3	1.00	0.31
		(−1.40 – 3.40)	

* Questions in Section 2 were about basic definitions while questions in Section 3 were about practical knowledge.

## Discussion

This study demonstrated increase in comfort level of all educational levels of participants after implementing a fairly simple and efficient ultrasound training program in a low-resource setting. Significant increase in basic and practical knowledge in medical student and junior house officer groups were observed, suggesting that the intervention is an effective teaching tool. This also demonstrated that novice and lower level trainees can efficiently learn practical skills that can aid in obstetric evaluation with instruction and hands-on practice.

In looking at the existing literature, training with handson approach resulted in improved self-reported comfort and increase in knowledge of basic ultrasound concepts. Lee et al surveyed residents and program directors during the 2003 Council on Resident Education in Obstetrics and Gynecology (CREOG) exam regarding fetal ultrasound training for obstetrics and gynecology residents and found that hands-on scanning and observation were the most effective educational activities for ultrasound training.^16^

Studies evaluating training programs in low-resource areas consistently demonstrate efficacy in developing proficiency in basic ultrasound skills among non-specialist providers. 3–10,12–13 A six-week focused training in ultrasound for the midwives at a clinic in rural Uganda was evaluated by Swanson et al, demonstrating high specificity and sensitivity in detection of twins and fetal presentation.^10^ Greenwold et al examined an eight-week training course with subsequent ten-month remote training that was used in four antenatal clinics in rural Mozambique. After completion of the training, they found similar detection rates of twin pregnancy, malpresentation, and placenta previa between the group in which a trainee was scanning with direct supervision of an expert and the trainee group that was scanning alone.^3^ Stein et al developed a two-month training program to prepare midwives to be the first line ultrasound providers in a two-level ultrasound service in Tanzania. They demonstrated 100% matching between reported results by trained midwives and specialist sonographers in the identification of multiple pregnancies, fetal heartbeat, and fetal position.^6^

The relevance of training providers in ultrasound is supported by studies that assess applications of routine and diagnostic ultrasound examinations. According to a meta-analysis of 11 control trials published in the Cochrane Library, there are numerous benefits in performing routine ultrasound prior to 24 weeks gestation.^17^ Early dating ultrasounds have been shown to independently decrease induction rates for “postdates” pregnancies from 10% to 1.5%. They are also useful in diagnosing multiple pregnancies, and determining chorionicity. Growth assessment, fetal presentation, placental position and diagnosis of intrauterine fetal demise can also help guide management of pregnancy and delivery.^17^

The application, benefit, and risk of more widely available ultrasound evaluation in low-income settings have been discussed previously in the literature.^2^–^6^,^9^,^10^,^15^,^18^,^19^ While routine ultrasound in resource-limited and developing areas has not been found to improve maternal or fetal mortality, several studies^2^,^3^,^18^ have shown a reduction in frequency of referrals to tertiary care centers by enabling these rural centers to rule out ruptured membranes and even more dangerous complications such as placenta previa or malpresentation. The literature also suggests the potential of improved accuracy of pregnancy dating, if these initial biometry scans can be performed earlier, before the patient is able to travel to a larger center for detailed scans.

Seffah et al reviewed the utility of obstetric ultrasound in developing countries. The cost (in US dollars) of a single 2-dimensional scan in developing countries was reported to be $1.30. They also raise several points regarding limitations including frequent power-outages, lack of customized growth charts for the local population, and absence of standardized training.^2^

Ross et al analyzed compliance with antenatal clinic visits when routine screening ultrasound was employed for high-risk pregnancies in a community clinic in rural Uganda. They found an increase in both number of antepartum visits and attended deliveries at the clinic after ultrasound was incorporated into the antepartum visits. This suggests that use of routine ultrasound may also encourage women with high-risk pregnancies to seek skilled obstetric health care.^20^ This is particularly relevant in Uganda given that it is estimated that facilities with emergency obstetric services only see about 5% of the births occurring in the country.^21^

The overuse and misuse of ultrasound has also been observed in prior studies, including excessive self-referral in self-pay systems, false gender assignment, and gender selective abortions. These implications must be considered when training sonographers and counseling patients based on ultrasound results.^2^,^3^,^19^

Our study had several limitations. A relatively small number of SHO participants were able to participate in the study due to exams during the study period. There was no control group and the time interval between pre- and post-testing was not standardized; potentially resulting in variation in the amount of practice or retention between individuals. The overall training period was very short. This study was voluntary for those at the student level and therefore may have had an inherent selection bias for students interested in ultrasound.

Future research opportunities include expanding the scope of the study to include advanced obstetric and gynecologic ultrasound skills. Adding a control group would also aid in evaluating the efficacy of the intervention compared to real-time practice in the field without any formal teaching session. Additionally, the study population could be expanded to midwives and nurses. Ultimately, we hope this study will inspire more clinicians to develop and assess teaching tools in ultrasound training when working in developing areas of the world.
